# Iron deficiency: prevalence, mortality risk, and dietary relationships in general and heart failure populations

**DOI:** 10.3389/fcvm.2024.1342686

**Published:** 2024-03-18

**Authors:** Hui Sun, Qinhong Wang, Wenqiang Han, Changli Chen, Tianyu Wang, Jingquan Zhong

**Affiliations:** ^1^ Department of Cardiology, Qilu Hospital (Qingdao), Cheeloo College of Medicine, Shandong University, Qingdao, Shandong, China; ^2^ National Key Laboratory for Innovation and Transformation of Luobing Theory, The Key Laboratory of Cardiovascular Remodeling and Function Research, Chinese Ministry of Education, Chinese National Health Commission and Chinese Academy of Medical Sciences, Department of Cardiology, Qilu Hospital of Shandong University, Jinan, China

**Keywords:** iron deficiency, heart failure, ferritin, transferrin saturation, long-term prognosis, folate

## Abstract

**Background:**

Iron deficiency (ID) is the most common nutritional deficiency, with little research on its prevalence and long-term outcomes in the general population and those with heart failure (HF). Both the relationships between dietary iron and ID, as well as dietary folate and ID, are understudied.

**Methods:**

We used data from the National Health and Nutrition Examination Survey from 1999 to 2002 to investigate the prevalence, prognosis, and relationship between dietary and ID defined by different criteria in the general population (*n* = 6,660) and those with HF (*n* = 182).

**Results:**

There was no significant difference in the prevalence of ID between HF patients and the general population after propensity score matching. Transferrin saturation (TSAT) <20% was associated with higher 5-year all-cause mortality (HR: 3.49, CI: 1.40–8.72, *P* = 0.007), while ferritin <30 ng/ml was associated with higher 10-year (HR: 2.70, CI: 1.10–6.67, *P* = 0.031) and 15-year all-cause mortality (HR: 2.64, CI: 1.40–5.00, *P* = 0.003) in HF patients. Higher dietary total folate but dietary iron reduced the risk of ID (defined as ferritin <100 ng/ml) in HF patients (OR: 0.80; 95% CI: 0.65–1.00; *P* = 0.047).

**Conclusions:**

The prevalence of ID was identical in HF and non-HF individuals. Ferritin <30 ng/ml was associated with long-term outcomes whereas TSAT <20% was associated with short-term prognosis in both the general population and HF patients. A diet rich in folate might have the potential for prevention and treatment of ID in HF patients.

## 1 Introduction

Iron deficiency (ID) with or without anemia is a common disease worldwide that is associated with impaired exercise capacity and mortality (1, 2). The prevalence of ID in individuals with heart failure (HF) ranges from 37% to 75.3%, while in the general population, it varies from 16.4% to 64.3% (3–6). These prevalence rates are contingent upon the particular definition of ID employed and the specific demographic under investigation. Nevertheless, literature directly comparing the incidence of ID between these two cohorts is notably lacking.

ID has been defined in various ways (as defined by heart failure guideline criteria: ferritin <100 ng/ml or, when 100–299 ng/ml, a transferrin saturation (TSAT) <20%; ferritin <15 ng/ml; ferritin <30 ng/ml; ferritin <100 ng/ml; TSAT <20%; or serum iron <13 μmol/L) (1, 14), prior research has primarily examined the association between isolated or a few definitions of ID and mortality. Moreover, research efforts have predominantly concentrated on the link between ID and short-term prognosis, leaving a gap in our understanding of its impact on long-term prognostic outcomes. In instances where certain definitions of ID are subject to scrutiny (7, 11, 15), a thorough assessment of the relationship between widely accepted definitions of ID and both short-term and long-term prognoses is imperative.

In certain research studies, a notable and statistically significant positive association between ferritin levels and iron intake was evident (16, 17). Inadequate iron consumption emerged as a noteworthy predictor for maintaining serum ferritin levels below 30 ng/ml, though not for achieving ferritin concentrations <100 ng/ml (16). The accuracy of these findings necessitates validation within a more expansive and diverse population sample. Additionally, it is imperative to determine their applicability, especially among individuals affected by HF. Moreover, folate, a vital determinant of hematological status, is advocated for concurrent use with iron to expedite the resolution of ID anemia, particularly during pregnancy (18, 19). However, the association between dietary folate and ID remains uncertain both within the general population and among individuals with HF.

We aimed to investigated the prevalence of ID defined by all the aforementioned definitions in both the general population and HF individuals. Besides, the association of ID with long-term outcomes was explored. We finally studied how dietary iron and total folate are linked to ID to find potential ways for ID prevention and treatment.

## 2 Methods

### 2.1 Study population

The publicly available data were collected from the 1999–2002 cycle of the National Health and Nutrition Examination Survey (NHANES), which is a program of studies designed to assess the health and nutritional status of adults and children in the United States. Each year, the survey assesses a sample of approximately 5,000 individuals from counties across the country, with 15 counties visited annually. NHANES is a significant program under the auspices of the National Center for Health Statistics (NCHS), which is a division of the Centers for Disease Control and Prevention (CDC), and is responsible for generating critical health and vital statistics for the country (20). Participants who had complete information on demographics, medical history (congestive heart failure, coronary heart disease, stroke, cancer, hypertension, diabetes and hospitalization), specific laboratory measurements (NT-proBNP, hemoglobin, serum iron, ferritin, transferrin saturation, creatinine, alanine aminotransferase, aspartate aminotransferase, C-reactive protein, segmented neutrophils percent and glycohemoglobin), dietary iron and total folate and corresponding sample weights were included. Of the 6,660 participants, 182 had HF. The survey protocol was approved by the NCHC (National Center for Health Statistics) Ethics Review Board and all participants provided informed written consent (21). All methods were carried out in accordance with Declaration of Helsinki.

### 2.2 Derived variables

The glomerular filtration rate was estimated by the CKD-EPI equation (22). In this study, the third quartile of the percentage of neutrophils in the general population was 64.6%. Based on this value, both the general population and HF patients were divided into high and low neutrophil percentage groups. In the present study, the median daily intake of iron and total folate in the general population were 13.42 mg and 345 mcg, respectively. Based on these values, the population was divided into high and low intake groups. Anemia was diagnosed in males with hemoglobin levels below 13 g/dl and females with hemoglobin levels below 12 g/dl. Participants with a ratio of family income to poverty less than 1.3 were classified as low-income group, while those with a ratio greater than or equal to 1.3 were classified as middle-to-high income group. According to the Physical Activity Questionnaire which was based on the Global Physical Activity Questionnaire and included questions related to daily activities, leisure time activities, and sedentary activities, the classification of cardiac function was divided into two groups: NYHA I/II and NYHA III/IV.

### 2.3 Dietary data and sample weights

The in-person interview for participants was conducted in a private room in the NHANES mobile examination center in the United States to collect dietary data during the 24-h period prior to the interview. Some of the NHANES 1999–2000 dietary interview data were collected by a telephone dietary interview. In 2001, dietary intake data were collected using the NHANES computer-assisted dietary interview system, which was also used to collect dietary data for the 1999–2000 collection period. In 2002, data were collected using USDA's dietary data collection instrument, the Automated Multiple Pass Method. The sample weights Weighted Dietary Recall 4-Year (WTDR4YR) which were constructed by taking the Mobile Examination Center sample weights (WTMEC2YR) and further adjusting for the additional non-response and the differential allocation by day of the week for the dietary intake data collection were applied to produce national, representative estimates.

### 2.4 Survival data

The National Center for Health Statistics has linked data collected from several NCHS population surveys with death certificate records from the National Death Index. The public-use LMF provide mortality follow-up data from the date of survey participation through December 31, 2019 (23).

### 2.5 Statistical analysis

The statistical analysis was performed using the R programming language (version 4.1.3). Continuous variables were presented as Median (IQR). Categorical variables were presented as *n* (unweighted) (%). To compare the baseline characteristics, the Wilcoxon rank-sum test for complex survey samples and the chi-squared test with Rao & Scott's second-order correction were used. Propensity score matching (PSM) was utilized to eliminate bias and control for demographics (age, sex, race, income and BMI) using the “MatchIt” package. The weighted Cox proportional hazards models and weighted logistic regression models were performed using the “survey” package. Variables with *P* ≤ 0.1 in univariable models were included in the multivariable models. Significance was defined as two-sided probability values less than 0.05.

## 3 Results

### 3.1 Baseline characteristics

**Figure 1:**
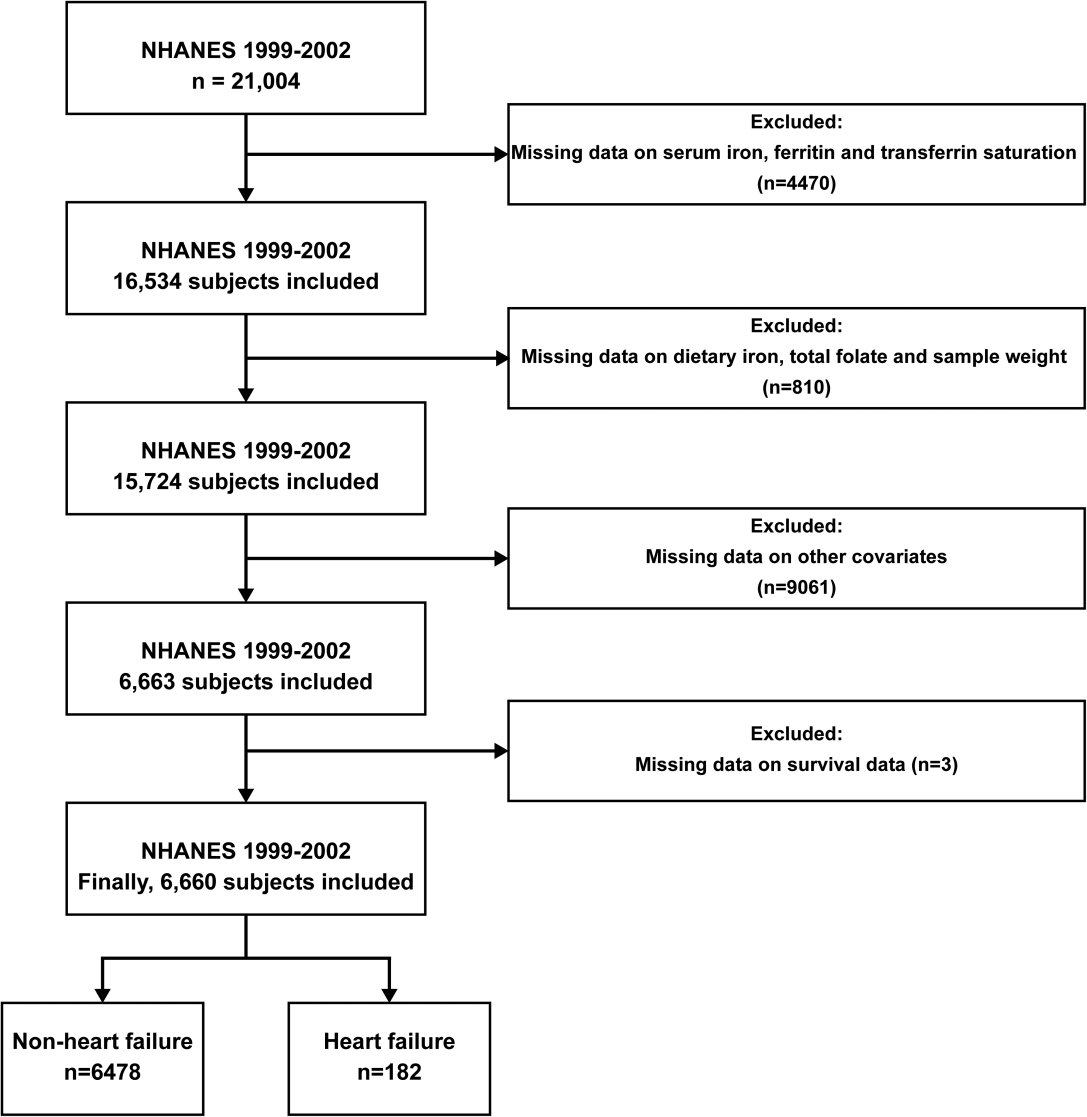
Flow chart.

During the period of 1999–2002, the NHANES database documented data from 21,004 individuals. However, only 6,663 individuals had complete baseline information, and an additional 3 individuals did not have survival data. Among the remaining 6,660 individuals, 182 self-reported that they had congestive heart failure (Figure 1).

For both HF patients and non-HF individuals, there were no significant differences in their iron indices (serum ferritin (99 vs. 85 ng/ml, *P* = 0.2), serum iron (14 vs. 15 μmol/L, *P* = 0.088) and TSAT (21% vs. 24%, *P* = 0.12) (Table 1). Besides, after adjusting for age, gender, income, and BMI using propensity score matching, there was no significant difference in the incidence of ID (as defined by all the aforementioned definitions) between individuals with HF and those without (Supplementary Table S1).

**Table 1:** Baseline characteristics of heart failure and non-heart failure individuals.

	All* (*N* = 6,660)	Non-heart failure* (*n* = 6,478)	Heart failure* (*n* = 182)	*P*-value^†^
Age, year	44 (33, 58)	44 (32, 57)	65 (53, 76)	<0.001
Male	3,162 (48%)	3,061 (48%)	101 (52%)	0.396
Race/ethnicity				0.340
Mexican American	1,567 (6.8%)	1,531 (6.9%)	36 (3.0%)	
Other Hispanic	350 (6.3%)	342 (6.3%)	8 (5.9%)	
Non-Hispanic White	3,413 (74%)	3,312 (73%)	101 (78%)	
Non-Hispanic Black	1,122 (9.3%)	1,089 (9.3%)	33 (11%)	
Other Race	208 (4.1%)	204 (4.1%)	4 (2.4%)	
Poverty income ratio < 1.3	1,799 (21%)	1,722 (20%)	77 (45%)	<0.001
BMI, kg/m^2^				0.004
<18	62 (1.3%)	60 (1.3%)	2 (0.4%)	
18–25	2,015 (33%)	1,978 (33%)	37 (19%)	
25–30	2,438 (35%)	2,369 (35%)	69 (38%)	
≥30	2,145 (31%)	2,071 (30%)	74 (42%)	
NYHA functional class III/IV			69 (41%)	
Medical history				
Coronary heart disease	267 (3.6%)	188 (2.7%)	79 (41%)	<0.001
Stroke	180 (2.2%)	148 (1.9%)	32 (16%)	<0.001
Hypertension	1,973 (26%)	1,848 (25%)	125 (61%)	<0.001
Diabetes	601 (6.4%)	552 (6.0%)	49 (22%)	<0.001
Cancer	536 (8.2%)	498 (7.7%)	38 (28%)	<0.001
Anemia	532 (5.1%)	505 (5.0%)	27 (11%)	0.011
Treatment for anemia (past 3 months)	217 (2.7%)	199 (2.6%)	18 (10%)	<0.001
Hospitalization (past 12 months)	791 (11%)	729 (10%)	62 (34%)	<0.001
Laboratory measurements				
NT-proBNP, pg/ml	46 (22, 95)	45 (22, 90)	285 (116, 742)	<0.001
Hemoglobin, g/dl	14.40 (13.50, 15.40)	14.50 (13.50, 15.40)	13.95 (13.20, 15.32)	0.042
C-reactive protein, mg/dl	0.21 (0.08, 0.47)	0.21 (0.08, 0.46)	0.41 (0.16, 0.96)	<0.001
Percentage of neutrophils >64.6%	1,812 (25%)	1,753 (25%)	59 (35%)	0.036
ALT, U/L	21 (16, 29)	21 (16, 29)	20 (15, 27)	0.256
AST, U/L	22 (19, 26)	22 (19, 26)	22 (19, 26)	0.668
eGFR, ml/min/1.73 m^2^	104 (89, 117)	104 (90, 118)	83 (56, 97)	<0.001
Glycohemoglobin, %	5.30 (5.00, 5.50)	5.30 (5.00, 5.50)	5.50 (5.30, 6.28)	<0.001
Serum iron, μmol/L	15 (11, 20)	15 (11, 20)	14 (10, 20)	0.088
Ferritin, ng/ml	85 (38, 165)	85 (38, 165)	99 (52, 181)	0.158
Transferrin saturation, %	24 (17, 31)	24 (18, 31)	21 (16, 29)	0.118
ID (heart failure guideline criteria)	4,305 (66%)	4,185 (66%)	120 (66%)	>0.9
Ferritin <300 ng/ml	6,016 (92%)	5,852 (92%)	164 (94%)	0.287
Ferritin <100 ng/ml	3,696 (56%)	3,605 (57%)	91 (50%)	0.187
Ferritin <30 ng/ml	1,327 (19%)	1,302 (20%)	25 (15%)	0.383
Ferritin <15 ng/ml	614 (7.8%)	604 (7.9%)	10 (3.3%)	0.032
TSAT <20%	2,431 (35%)	2,350 (35%)	81 (45%)	0.040
Serum iron ≤13 μmol/L	2,463 (35%)	2,378 (35%)	85 (44%)	0.110
Serum iron ≤10 μmol/L	1,205 (17%)	1,160 (16%)	45 (27%)	0.034

ALT, alanine transaminase; AST, aspartate transaminase; eGFR, estimated glomerular filtration rate; ID, iron deficiency; NT-proBNP, N-terminal pro-B-type natriuretic peptide; NYHA, new york heart association; TSAT, transferrin saturation.

* Median (IQR); *n* (unweighted) (%).

^†^ Wilcoxon rank-sum test for complex survey samples; chi-squared test with Rao & Scott's second-order correction.

### 3.2 Five-, ten-, and fifteen-year all-cause mortality of ID in general population

Among the cohort of 6,660 individuals, 373 decease cases were recorded after a 5-year follow-up, followed by 899 mortalities at the 10-year mark, and 1,416 mortalities at the 15-year follow-up period.

#### 3.2.1 Five-year outcome of iron deficiency

In general population recorded in NHANES 1999–2002, after adjusting for confounding factors, there was no significant association between ID, as defined by various methods, and five-year all-cause mortality in the general population (Supplementary Table S2). However, subgroup analysis revealed that in the general population aged 45 years and above, TSAT <20% was associated with an increased risk of all-cause mortality at 5 years (HR: 1.40; 95% CI: 1.05–1.86; *P* = 0.021), as well as ferritin <30 ng/ml (HR: 1.85; 95% CI: 1.18–2.89; *P* = 0.007) (Supplementary Table S3).

#### 3.2.2 Ten-year outcome of iron deficiency

In adjusted models, only ferritin <30 ng/ml was significantly associated with 10-year all-cause mortality (HR: 1.35; 95% CI: 1.03–1.77; *P* = 0.030, Supplementary Table S4), particularly among individuals aged 45 years and above (HR: 1.47; 95% CI: 1.14–1.89; *P* = 0.003, Supplementary Table S3). Alternative definitions for ID were found to be inadequate in predicting 10-year all-cause mortality (Supplementary Tables S3, S4).

#### 3.2.3 Fifteen-year outcome of iron deficiency

In Univariable analysis, ID defined by criteria in guideline for heart failure (12) (HR: 0.69; 95% CI: 0.61–0.78; *P* < 0.001), ferritin <300 ng/ml (HR: 0.62; 95% CI: 0.49–0.79; *P* < 0.001), ferritin <100 ng/ml (HR: 0.66; 95% CI: 0.57–0.76; *P* < 0.001), ferritin <30 ng/ml (HR: 0.48; 95% CI: 0.38–0.60; *P* < 0.001), ferritin <15 ng/ml (HR: 0.42; 95% CI: 0.30–0.59; *P* < 0.001) were significantly associated with the 15-year all-cause mortality while serum iron (≤10 μmol/L or ≤13 μmol/L) and TSAT <20% were not (Supplementary Table S5). After adjusting for confounding factors, ferritin levels, iron levels or TSAT was not associated with 15-year all-cause mortality (Supplementary Tables S3, S5).

### 3.3 Five-, ten-, and fifteen-year all-cause mortality of ID in heart failure patients

Within the group of 182 patients diagnosed with HF, 46 fatalities were recorded during the 5-year follow-up, followed by 94 mortalities at the 10-year follow-up, and 126 mortalities at the 15-year follow-up.

#### 3.3.1 Five-year outcome of iron deficiency

In unadjusted analyses, ID defined by serum iron levels (≤10 μmol/L: HR: 3.32; 95% CI: 1.83–6.03; *P* < 0.001; ≤13 μmol/L: HR: 3.08; 95% CI: 1.64–5.77; *P* < 0.001), TSAT <20% (HR: 3.96; 95% CI: 1.93–8.12; *P* < 0.001) and ferritin <30 ng/ml (HR: 2.73; 95% CI: 1.24–6.02; *P* = 0.013) were associated with higher 5-year all-cause mortality (Table 2). In adjusted models, TSAT <20% (HR: 3.49; 95% CI: 1.40–8.72; *P* = 0.007) and ferritin <300 ng/ml (HR: 4.79; 95% CI: 1.43–16.0; *P* = 0.011) were significantly associated with higher 5-year all-cause mortality. ID defined by heart failure guideline criteria (HR: 2.52; 95% CI: 0.92–6.90; *P* = 0.073) or iron ≤10 μmol/L (HR: 2.17; 95% CI: 0.97–4.83; *P* = 0.059) seemed to associated with a higher mortality, but not significant (Table 2).

**Table 2:** Cox proportional hazards model for 5-year all-cause mortality (HF, *n* = 182).

	Univariable model			Multivariable model*		
	HR	95% CI	*P*-value	HR	95% CI	*P*-value
ID (heart failure guideline criteria)	2.14	0.98, 4.70	0.057	2.52	0.92, 6.90	0.073
Ferritin <300 ng/ml	1.05	0.30, 3.70	>0.9	4.79	1.43, 16.0	0.011
Ferritin <100 ng/ml	1.45	0.68, 3.08	0.334	1.12	0.48, 2.63	0.795
Ferritin <30 ng/ml	2.73	1.24, 6.02	0.013	2.51	0.75, 8.36	0.133
Ferritin <15 ng/ml	2.97	0.86, 10.3	0.086	2.73	0.64, 11.6	0.176
TSAT <20%	3.96	1.93, 8.12	<0.001	3.49	1.40, 8.72	0.007
Serum iron ≤13 μmol/L	3.08	1.64, 5.77	<0.001	1.82	0.91, 3.67	0.093
Serum iron ≤10 μmol/L	3.32	1.83, 6.03	<0.001	2.17	0.97, 4.83	0.059

ALT, alanine transaminase; CI, confidence interval; ID, iron deficiency; HF, heart failure; HR, hazard ratio; NT-proBNP, N-terminal pro-B-type natriuretic peptide; NYHA, new york heart association; TSAT, transferrin saturation.

* Model adjusted for age (/5), ln(NT-proBNP), estimate glomerular filtration rate, body mass index, NYHA functional class.

#### 3.3.2 Ten-year and fifteen-year outcome of iron deficiency

Ferritin <30 ng/ml (10-year: HR: 2.83; 95% CI: 1.67–4.80; *P* < 0.001, Table 3, and 15-year: HR: 2.45; 95% CI: 1.40–4.30; *P* = 0.002, Supplementary Table S6) significantly associated with 10-year and 15-year all-cause mortality, even after adjusting confounding factors (10-year HR: 2.70; 95% CI: 1.10–6.67; *P* = 0.031, Table 3, and 15-year: HR: 2.64; 95% CI: 1.40–5.00; *P* = 0.003, Supplementary Table S6).

**Table 3:** Cox proportional hazards model for 10-year all-cause mortality (HF, *n* = 182).

	Univariable model			Multivariable model*		
	HR	95% CI	*P*-value	HR	95% CI	*P*-value
ID (heart failure guideline criteria)	1.54	0.89, 2.69	0.125	1.35	0.68, 2.67	0.390
Ferritin <300 ng/ml	1.37	0.47, 4.01	0.570	1.79	0.61, 5.22	0.288
Ferritin <100 ng/ml	1.51	0.98, 2.32	0.062	1.08	0.60, 1.94	0.804
Ferritin <30 ng/ml	2.83	1.67, 4.80	<0.001	2.70	1.10, 6.67	0.031
Ferritin <15 ng/ml	2.45	0.98, 6.11	0.055	2.37	0.89, 6.29	0.084
TSAT <20%	1.52	0.77, 3.00	0.224	1.43	0.74, 2.77	0.289
Serum iron ≤13 μmol/L	1.84	0.94, 3.62	0.077	1.28	0.68, 2.43	0.448
Serum iron ≤10 μmol/L	2.05	0.95, 4.39	0.066	1.64	0.79, 3.40	0.184

ALT, alanine transaminase; CI, confidence interval; HF, heart failure; HR, hazard ratio; ID, iron deficiency; NT-proBNP, N-terminal pro-B-type natriuretic peptide; TSAT, transferrin saturation.

* Model adjusted for age (/5), gender, body mass index, anemia, cancer, ln(ALT), ln(NT-proBNP), estimate glomerular filtration rate, percentage of neutrophils.

### 3.4 Associations between dietary intake and incidence of iron deficiency

In the general population, the median daily intake of iron is 13.42 mg, while the median daily intake of total folate is 345 mcg. Based on the median intake, the entire population was categorized into high and low intake groups.

#### 3.4.1 General population

Through univariable logistic regression analysis, a correlation was found between a higher intake of dietary iron and a decreased risk of ID. However, when gender was included in the multivariable regression analysis, no significant relationship was observed between dietary iron intake and ID (Supplementary Table S7).

Except when ID was defined as ferritin <300 ng/ml, ferritin <15 ng/ml, or iron <13 μmol/L, univariable regression analysis showed a significant relationship between dietary total folate and ID. Despite this, when including gender in the multivariable regression analysis, there was no significant association between the dietary intake of total folate and any of the ID definitions (Supplementary Table S7).

#### 3.4.2 Heart failure patients

The analysis of HF patients revealed no significant relationship between dietary iron intake and ID in univariable logistic regression analysis. However, in multivariable logistic regression analysis, a higher intake of iron seemed to be associated with ferritin <15 ng/ml (OR: 1.08; 95% CI: 1.00–1.16; *P* = 0.050) (Supplementary Table S8).

In HF patients, the relationship between total folate intake and ID was significant when ID was defined as ferritin <30 ng/ml. A higher intake of total folate was associated with a higher risk of ID, as shown by both univariable (OR: 1.19; 95% CI: 1.03–1.38; *P* = 0.023) and multivariable regression analyses (excluding gender: OR: 1.21; 95% CI: 1.04–1.41; *P* = 0.015, and including gender: OR: 1.21; 95% CI: 1.05–1.39; *P* = 0.009) (Supplementary Table S8).

Following the exclusion of HF patients with ferritin <30 ng/ml, a re-analysis of the remaining subjects revealed a potential association between higher levels of dietary total folate intake and the lower risk of ID, as defined by ferritin <100 ng/ml (univariable: OR: 0.78; 95% CI: 0.63–0.96; *P* = 0.022, multivariable (excluding gender): OR: 0.80; 95% CI: 0.65–0.99; *P* = 0.042 and multivariable (including gender): OR: 0.80; 95% CI: 0.65–1.00; *P* = 0.047) (Table 4).

**Table 4:** Relationship of dietary intake to iron deficiency in HF patients with ferritin ≥30 ng/ml (logistic regression model).

		Model 1			Model 2*			Model 3^†^		
Group	Dietary intake	OR	95% CI	*P*-value	OR	95% CI	*P*-value	OR	95% CI	*P*-value
Ferritin <100 ng/ml	Iron	1.01	0.77, 1.32	>0.9						
	Total folate	0.78	0.63, 0.96	0.022	0.80	0.65, 0.99	0.042	0.80	0.65, 1.00	0.047
ID (heart failure guideline criteria)	Iron	0.96	0.71, 1.30	0.781						
	Total folate	0.83	0.60, 1.15	0.249						
Ferritin <300 ng/ml	Iron	0.95	0.85, 1.07	0.398						
	Total folate	0.91	0.79, 1.04	0.146						
TSAT <20%	Iron	0.99	0.80, 1.23	>0.9						
	Total folate	1.02	0.76, 1.37	0.893						
Serum iron ≤13 μmol/L	Iron	1.10	0.90, 1.36	0.334						
	Total folate	1.01	0.78, 1.32	>0.9						
Serum iron ≤10 μmol/L	Iron	1.17	0.95, 1.45	0.137						
	Total folate	1.12	0.88, 1.43	0.337						

ALT, alanine transaminase; AST, aspartate transaminase; CI, confidence interval; HF, heart failure; ID, iron deficiency; OR, odds ratio; TSAT, transferrin saturation.

* Model 2: Model adjusted for race, ln (ALT), ln (AST), hemoglobin.

^†^ Model 3: Model 2 + gender.

## 4 Discussion

In the present investigation, we substantiated that the occurrence of ID was consistent between individuals in the general population and those diagnosed with HF. Moreover, a ferritin level below 30 ng/ml was linked to a higher risk of all-cause mortality over 10 and 15 years in HF patients, and similarly, it was associated with a 10-year all-cause mortality in the general population. Furthermore, a reduced dietary intake of total folate emerged as a significant predictive factor for maintaining serum ferritin levels below 100 ng/ml specifically in HF patients.

### 4.1 Prevalence of iron deficiency in patients with heart failure and non-heart failure individuals

From the data of NHANES III, the prevalence of ID (defined by heart failure guideline criteria) in 574 participants (male: 50.2%) with self-reported HF was 61.3% (5). In a prospective observational study of 546 patients (male: 88%) with stable systolic chronic HF, the prevalence of ID (defined by heart failure guideline criteria) was 37% in the entire population of systolic chronic HF patients (4). In a survey of 832 hospitalized patients with decompensated chronic HF, the prevalence of ID (defined by heart failure guideline criteria) was found to be 68.6% in male and 75.3% in female (3). In three European population-based cohorts (male: 45.2%), 60% of individuals had ferritin levels below 100 ng/ml, 16.4% had ferritin levels below 30 ng/ml, and 64.3% met the heart failure guideline criteria for ID (6). In the present study, the prevalence of ID defined by guideline criteria in HF patients (male: 52%) was 66%. It was noteworthy that the prevalence of ID was comparable in the non-HF population (Table 1, Supplementary Table S1), which contradicted our previous assumptions. A large proportion of HF patients had impaired nutritional status (24), which may affect iron metabolism. Besides, the proportion of anemia in HF patients is relatively high. These factors make us unconsciously believe that the proportion of ID in HF patients is relatively high. Alternatively, it was also hypothesized that HF patients would demonstrate a lower incidence of ID due to the pro-inflammatory state associated with HF, which could lead to elevated levels of ferritin (25, 26). In terms of disease prevalence, the existing diagnostic criteria for ID are ineffective in distinguishing between individuals with HF and those without.

### 4.2 Prognostic value of iron deficiency

In line with the findings reported by Masini et al. (7), our study revealed a significant association between TSAT <20% and all-cause mortality over a five-year period in HF patients, whereas levels of ferritin did not demonstrate a significant association (Table 2). However, our findings demonstrated a significant association between low levels of ferritin (<30 ng/ml) and an elevated risk of long-term all-cause mortality in both HF patients and the general population. Hence, TSAT emerges as a predictor of short-term all-cause mortality in HF patients, whereas ferritin demonstrates greater suitability for predicting long-term all-cause mortality, possibly due to the inherent fluctuations of TSAT and ferritin levels in the human body. In patients with HF, 55.5% of patients with iron ≤13 μmol/L at baseline remained ID after 1 year. Applying TSAT <20% as the definition of ID, 58.2% of patients remained ID after 1 year. When applying the heart failure guideline criteria, this proportion rose to 79.4% (27). Of the 537 patients with baseline ID (TSAT <20%), 53% (284 individuals) still had ID after 6 months. Meanwhile, 20% (128 individuals) of the 451 patients without baseline ID developed ID by the 6-month follow-up (15). Another possible reason is that the definitions of ID mentioned above were overly inclusive, encompassing a substantial proportion of individuals who did not truly exhibit ID. Therefore, the observed changes in iron status may have been only normal fluctuations within the normal range. A randomized, placebo-controlled, double-blind trial (IronIC) found that heart transplant recipients with ID defined by heart failure guideline criteria had no improvement in peak oxygen consumption 6 months after intravenous iron treatment while the peak oxygen consumption was significantly improved in patients with ferritin <30 ng/ml at baseline (4.4 ml/kg/min, 95% confidence interval 0.01–8.72, *P* = 0.0495) (28). Hence, a diagnostic threshold of ferritin <30 ng/ml may be more appropriate, as it is associated with the prognosis of both HF and non-HF individuals. Nevertheless, additional research and validation are warranted to investigate the stability of iron status in individuals presenting ferritin levels below this threshold.

In the present study, it is recommended that when the ferritin level is <30 ng/ml or the TSAT level is <20%, treatment should be considered for the general population aged 45 years and above. For patients with HF, intravenous iron replacement therapy should be considered when the ferritin level is <30 ng/ml (15%) or the TSAT level is <20% (45%). In our study, a ferritin level <300 ng/ml is also associated with all-cause mortality in patients with HF over a period of five years, therefore, a ferritin level >300 ng/ml may be used as a treatment target, and revious research has demonstrated the feasibility of achieving a ferritin level >400 ng/ml through intravenous iron replacement therapy (8).

### 4.3 Iron deficiency, dietary iron, and total folate

Iron from dietary sources exists in two primary forms: haem iron and non-haem iron. Haem iron is absorbed from the gastrointestinal tract with notably higher efficiency (29). Notably, the consumption of haem iron, rather than total iron intake, exhibits a significant correlation with ferritin levels (16, 17). Extensive research has demonstrated the enhanced effectiveness of a combination of folic acid and iron, as opposed to iron supplementation alone, in addressing ID (18, 19). In the present study, after adjusting for confounding factors such as gender, there was no significant relationship between dietary iron intake and ID. It is not surprising that there is no significant association between dietary iron intake and ID when oral iron therapy failed to achieve the desired effect (30). However, for HF patients, after adjusting for covariates such as anemia, gender and anemia-related treatment, a significant correlation was observed between higher total folate intake and an increased risk of ferritin <30 ng/ml. That might be explained by the tendency of HF patients with low levels of ferritin to receive recommendations or to spontaneously increase their consumption of iron and folate-rich foods. After excluding these severe ID patients (ferritin <30 ng/ml), it is promising that an increase in the consumption of folate-rich foods seems to contribute to a reduction in the incidence of ID (ferritin <100 ng/ml, OR: 0.80; 95% CI: 0.65–1.00; *P* = 0.047). Given the suboptimal outcomes observed in the treatment of ID in HF patients using oral iron supplementation, oral folic acid may represent a promising avenue for intervention (30). However, it is imperative to elucidate the underlying mechanism through which increased folate intake mitigates iron deficiency, without considering iron intake, warranting further exploration.

## 5 Limitations

This study has several limitations. Firstly, out of the total 21,004 participants, only a subset underwent testing for ferritin and other related measures, and not all selected individuals were able to comply with the scheduled appointments. However, NHANES provided sample weights to correct for unequal sampling probabilities and non-participation of selected individuals, ensuring that the final results were representative of the national population. Secondly, heart failure status in the NHANES database relied on self-reported participant responses through questionnaires, potentially introducing self-report bias. Meanwhile, there was a dearth of heart failure classification data. Thirdly, the cohort of patients with HF was relatively small, which presented a significant numerical disparity compared to the non-HF group and raised notable concerns. Fourthly, while this study analyzed neutrophil percentage and C-reactive protein as confounding factors, it's important to recognize that the complex interplay between ID, inflammation, ferritin, and iron metabolism could potentially influence the research conclusions. In addition, the paradoxical correlation between total folate intake and severe iron deficiency (ferritin <30 ng/ml) in the present study requires further investigation, as our assumption lacks supporting evidence. Furthermore, the results of this study need to be validated in further prospective research with larger sample sizes. Dynamic monitoring of iron status and dietary data should be conducted, and there was also a lack of measurement data for soluble transferrin receptor.

## 6 Conclusions

The prevalence of iron deficiency was identical in heart failure and non-heart failure individuals. Ferritin <30 ng/ml was associated with the long-term outcome of both the general and heart failure populations, while transferrin saturation (TSAT) <20% was associated with the 5-year mortality of heart failure patients and the relatively healthy population aged 45 years and above. A diet rich in folate might have the potential for prevention and treatment of iron deficiency in heart failure patients.

## Data Availability

The original contributions presented in the study are included in the article/Supplementary Material, further inquiries can be directed to the corresponding author.
