# Supplementary material for: Iron deficiency: prevalence, mortality risk, and dietary relationships in general and heart failure populations
Source: Front Cardiovasc Med. 2024 Mar 18;11:1342686. doi: 10.3389/fcvm.2024.1342686 (PMC10982413; doi:10.3389/fcvm.2024.1342686)
Supplement: Supplementary file 1 [file Table1.doc]

**Supplemental Material**

**Iron Deficiency: Prevalence, Mortality Risk, and Dietary Relationships in General and Heart Failure Populations**

| **Contents** | **Page** |
| --- | --- |
| **Table S1.** Baseline Characteristics of Heart Failure and Non-Heart Failure Individuals After Propensity Score Matching. | **2-3** |
| **Table S2.** Cox Proportional Hazards Model for 5-year All-Cause Mortality (General Population, n = 6660) | **4** |
| **Table S3.** Cox Proportional Hazards Model for All-Cause Mortality Stratified by Age (General Population, n = 6660) | **5** |
| **Table S4.** Cox Proportional Hazards Model for 10-year All-Cause Mortality (General Population, n = 6660) | **6** |
| **Table S5.** Cox Proportional Hazards Model for 15-year All-Cause Mortality (General Population, n = 6660) | **7** |
| **Table S6.** Cox Proportional Hazards Model for 15-year All-Cause Mortality (HF, n=182) | **8** |
| **Table S7.** Relationship of Dietary Intake to Iron Deficiency in General Population (Logistic Regression Model) | **9-10** |
| **Table S8.** Relationship of Dietary Intake to Iron Deficiency in HF Patients (Logistic Regression Model) | **11-12** |

| **Table S1. Baseline Characteristics of Heart Failure and Non-Heart Failure Individuals After Propensity Score Matching.** | | | | |
| --- | --- | --- | --- | --- |
|  | **ALL ***  (N = 275) | **Non-heart failure ***  (n = 121) | **Heart failure ***  **(n = 154)** | **P-value †** |
| Age, y | 63 (51, 76) | 62 (48, 75) | 65 (53, 76) | 0.4 |
| Male | 202 (54%) | 102 (57%) | 100 (53%) | 0.6 |
| Race/ethnicity |  |  |  | 0.4 |
| Mexican American | 75 (3.6%) | 39 (4.3%) | 36 (3.0%) |  |
| Other Hispanic | 23 (6.2%) | 15 (6.5%) | 8 (6.0%) |  |
| Non-Hispanic White | 189 (75%) | 90 (72%) | 99 (77%) |  |
| Non-Hispanic Black | 61 (11%) | 28 (9.8%) | 33 (11%) |  |
| Other Race | 12 (4.7%) | 8 (7.5%) | 4 (2.4%) |  |
| Poverty income ratio <1.3 | 162 (43%) | 87 (42%) | 75 (45%) | 0.7 |
| BMI, kg/m^2^ |  |  |  | 0.7 |
| < 18 | 3 (0.5%) | 1 (0.5%) | 2 (0.4%) |  |
| 18 - 25 | 76 (21%) | 39 (22%) | 37 (20%) |  |
| 25 - 30 | 149 (40%) | 80 (43%) | 69 (38%) |  |
| ≥ 30 | 132 (39%) | 60 (35%) | 72 (42%) |  |
| ID (heart failure guideline criteria) | 219 (63%) | 101 (60%) | 118 (65%) | 0.4 |
| Ferritin <300 ng/mL | 316 (92%) | 154 (88%) | 162 (94%) | 0.12 |
| Ferritin <100 ng/mL | 167 (46%) | 78 (41%) | 89 (49%) | 0.2 |
| Ferritin <30 ng/mL | 42 (13%) | 17 (11%) | 25 (15%) | 0.4 |
| Ferritin <15 ng/mL | 17 (4.1%) | 7 (5.1%) | 10 (3.4%) | 0.5 |
| TSAT <20% | 140 (42%) | 61 (39%) | 79 (45%) | 0.5 |
| Serum iron ≤13 μmol/L | 156 (42%) | 73 (40%) | 83 (43%) | 0.7 |
| Serum iron ≤10 μmol/L | 80 (22%) | 36 (17%) | 44 (26%) | 0.13 |
|  |  |  |  |  |
| ***** Median (IQR); n (unweighted) (%) | | | | |
| **†** Wilcoxon rank-sum test for complex survey samples; chi-squared test with Rao & Scott's second-order correction | | | | |
| ID, iron deficiency; TSAT, transferrin saturation. | | | | |

| **Table S2. Cox Proportional Hazards Model for 5-year All-Cause Mortality (General Population, n = 6660)** | | | | | | |
| --- | --- | --- | --- | --- | --- | --- |
|  | **Univariable Model** | | | **Multivariable Model *** | | |
|  | **HR** | **95% CI** | **P-value** | **HR** | **95% CI** | **P-value** |
| ID (heart failure guideline criteria) | 0.70 | 0.51, 0.95 | 0.021 | 0.93 | 0.69, 1.24 | 0.6 |
| Ferritin <300 ng/mL | 0.71 | 0.53, 0.95 | 0.023 | 1.27 | 0.94, 1.72 | 0.11 |
| Ferritin <100 ng/mL | 0.61 | 0.47, 0.81 | <0.001 | 0.90 | 0.67, 1.21 | 0.5 |
| Ferritin <30 ng/mL | 0.80 | 0.53, 1.20 | 0.3 | 1.58 | 0.98, 2.57 | 0.063 |
| Ferritin <15 ng/mL | 0.59 | 0.34, 1.02 | 0.059 | 1.27 | 0.61, 2.62 | 0.5 |
| TSAT <20% | 1.38 | 1.05, 1.81 | 0.019 | 1.31 | 0.98, 1.74 | 0.066 |
| Serum iron ≤13 μmol/L | 1.53 | 1.17, 2.00 | 0.002 | 1.25 | 0.96, 1.62 | 0.094 |
| Serum iron ≤10 μmol/L | 1.60 | 1.14, 2.26 | 0.007 | 1.23 | 0.86, 1.77 | 0.3 |
| *****Model adjusted for age (/5), gender, race, poverty income ratio, coronary heart disease, congestive heart failure, stroke, hypertension, diabetes, cancer, anemia, hospitalization, ln(ALT), ln(AST), glycohemoglobin, ln(NT-proBNP), C-reactive protein, estimate glomerular filtration rate, percentage of neutrophils.  ALT, alanine transaminase; AST, aspartate transaminase; CI, confidence interval; HR, hazard ratio; ID, iron deficiency; NT-proBNP, N-terminal pro-B-type natriuretic peptide; TSAT, transferrin saturation. | | | | | | |

| **Table S3. Cox Proportional Hazards Model for All-Cause Mortality Stratified by Age (General Population, n = 6660)** | | | | | | | | | |
| --- | --- | --- | --- | --- | --- | --- | --- | --- | --- |
|  | **5-year** | | | **10-year** | | | **15-year** | | |
| **Characteristic** | **HR** | **95% CI** | **p-value** | **HR** | **95% CI** | **p-value** | **HR** | **95% CI** | **p-value** |
| **Age >44 years**  **(n = 3614)** |  |  |  |  |  |  |  |  |  |
| ID (heart failure guideline criteria) | 1.05 | 0.80, 1.38 | 0.7 | 0.91 | 0.79, 1.06 | 0.2 | 0.90 | 0.79, 1.03 | 0.14 |
| Ferritin <300 ng/mL | 1.19 | 0.91, 1.56 | 0.2 | 1.04 | 0.85, 1.27 | 0.7 | 1.01 | 0.81, 1.26 | >0.9 |
| Ferritin <100 ng/mL | 1.03 | 0.80, 1.33 | 0.8 | 0.95 | 0.81, 1.11 | 0.5 | 0.91 | 0.79, 1.06 | 0.2 |
| Ferritin <30 ng/mL | 1.85 | 1.18, 2.89 | 0.007 | 1.47 | 1.14, 1.89 | 0.003 | 1.17 | 0.94, 1.46 | 0.2 |
| Ferritin <15 ng/mL | 1.40 | 0.62, 3.18 | 0.4 | 1.14 | 0.70, 1.86 | 0.6 | 0.99 | 0.69, 1.40 | >0.9 |
| TSAT <20% | 1.40 | 1.05, 1.86 | 0.021 | 1.16 | 0.94, 1.44 | 0.2 | 1.05 | 0.87, 1.27 | 0.6 |
| Serum iron ≤13 μmol/L | 1.28 | 0.97, 1.70 | 0.086 | 0.97 | 0.80, 1.18 | 0.8 | 0.92 | 0.76, 1.11 | 0.4 |
| Serum iron ≤10 μmol/L | 1.20 | 0.88, 1.64 | 0.2 | 1.06 | 0.85, 1.34 | 0.6 | 0.95 | 0.73, 1.24 | 0.7 |
| **Age ≤44 years**  **(n = 3046)** |  |  |  |  |  |  |  |  |  |
| ID (heart failure guideline criteria) | 0.61 | 0.17, 2.15 | 0.4 | 1.12 | 0.41, 3.03 | 0.8 | 1.32 | 0.59, 2.97 | 0.5 |
| Ferritin <300 ng/mL | 1.55 | 0.34, 7.07 | 0.6 | 1.48 | 0.30, 7.30 | 0.6 | 1.43 | 0.45, 4.55 | 0.5 |
| Ferritin <100 ng/mL | 0.50 | 0.16, 1.62 | 0.2 | 0.92 | 0.36, 2.36 | 0.9 | 1.21 | 0.55, 2.64 | 0.6 |
| Ferritin <30 ng/mL | 1.16 | 0.31, 4.28 | 0.8 | 1.81 | 0.74, 4.44 | 0.2 | 0.79 | 0.37, 1.68 | 0.5 |
| Ferritin <15 ng/mL | 0.96 | 0.33, 2.80 | >0.9 | 1.87 | 0.66, 5.32 | 0.2 | 1.21 | 0.46, 3.17 | 0.7 |
| TSAT <20% | 1.08 | 0.37, 3.15 | 0.9 | 1.56 | 0.84, 2.88 | 0.2 | 1.56 | 0.91, 2.65 | 0.10 |
| Serum iron ≤13 μmol/L | 1.43 | 0.46, 4.50 | 0.5 | 1.30 | 0.75, 2.25 | 0.4 | 1.08 | 0.71, 1.64 | 0.7 |
| Serum iron ≤10 μmol/L | 1.99 | 0.53, 7.43 | 0.3 | 1.20 | 0.58, 2.47 | 0.6 | 0.99 | 0.51, 1.91 | >0.9 |
| HR, hazard ratio; ID, iron deficiency; TSAT, transferrin saturation. | | | | | | | | | |

| **Table S4. Cox Proportional Hazards Model for 10-year All-Cause Mortality (General Population, n = 6660)** | | | | | | |
| --- | --- | --- | --- | --- | --- | --- |
|  | **Univariable Model** | | | **Multivariable Model *** | | |
|  | **HR** | **95% CI** | **P-value** | **HR** | **95% CI** | **P-value** |
| ID (heart failure guideline criteria) | 0.68 | 0.59, 0.78 | <0.001 | 0.90 | 0.77, 1.05 | 0.2 |
| Ferritin <300 ng/mL | 0.63 | 0.50, 0.81 | <0.001 | 1.08 | 0.89, 1.32 | 0.4 |
| Ferritin <100 ng/mL | 0.64 | 0.54, 0.76 | <0.001 | 0.91 | 0.77, 1.08 | 0.3 |
| Ferritin <30 ng/mL | 0.62 | 0.45, 0.84 | 0.002 | 1.35 | 1.03, 1.77 | 0.030 |
| Ferritin <15 ng/mL | 0.49 | 0.33, 0.73 | <0.001 | 1.13 | 0.72, 1.78 | 0.6 |
| TSAT <20% | 1.16 | 0.99, 1.36 | 0.072 | 1.16 | 0.95, 1.42 | 0.15 |
| Serum iron ≤13 μmol/L | 1.17 | 1.01, 1.37 | 0.041 | 0.98 | 0.81, 1.17 | 0.8 |
| Serum iron ≤10 μmol/L | 1.26 | 1.00, 1.59 | 0.049 | 1.04 | 0.83, 1.31 | 0.7 |
| ***** Model adjusted for age (/5), gender, race, poverty income ratio, coronary heart disease, congestive heart failure, stroke, hypertension, diabetes, cancer, anemia, treatment for anemia, hospitalization, ln(ALT), ln(AST), glycohemoglobin, ln(NT-proBNP), C-reactive protein, estimate glomerular filtration rate, percentage of neutrophils.  ALT, alanine transaminase; AST, aspartate transaminase; CI, confidence interval; HR, hazard ratio; ID, iron deficiency; NT-proBNP, N-terminal pro-B-type natriuretic peptide; TSAT, transferrin saturation. | | | | | | |

| **Table S5. Cox Proportional Hazards Model for 15-year All-Cause Mortality (General Population, n = 6660)** | | | | | | |
| --- | --- | --- | --- | --- | --- | --- |
|  | **Univariable Model** | | | **Multivariable Model *** | | |
|  | **HR** | **95% CI** | **P-value** | **HR** | **95% CI** | **P-value** |
| ID (heart failure guideline criteria) | 0.69 | 0.61, 0.78 | <0.001 | 0.91 | 0.80, 1.04 | 0.2 |
| Ferritin <300 ng/mL | 0.62 | 0.49, 0.79 | <0.001 | 1.04 | 0.85, 1.28 | 0.7 |
| Ferritin <100 ng/mL | 0.66 | 0.57, 0.76 | <0.001 | 0.92 | 0.81, 1.04 | 0.2 |
| Ferritin <30 ng/mL | 0.48 | 0.38, 0.60 | <0.001 | 1.04 | 0.83, 1.30 | 0.8 |
| Ferritin <15 ng/mL | 0.42 | 0.30, 0.59 | <0.001 | 0.97 | 0.69, 1.36 | 0.8 |
| TSAT <20% | 1.04 | 0.90, 1.20 | 0.6 | 1.08 | 0.89, 1.31 | 0.4 |
| Serum iron ≤13 μmol/L | 1.07 | 0.94, 1.22 | 0.3 | 0.93 | 0.77, 1.11 | 0.4 |
| Serum iron ≤10 μmol/L | 1.09 | 0.89, 1.33 | 0.4 | 0.95 | 0.73, 1.22 | 0.7 |
| ***** Model adjusted for age (/5), gender, race, poverty income ratio, body mass index, coronary heart disease, congestive heart failure, stroke, hypertension, diabetes, cancer, anemia, treatment for anemia, hospitalization, ln(ALT), ln(AST), glycohemoglobin, ln(NT-proBNP), C-reactive protein, estimate glomerular filtration rate, percentage of neutrophils.  ALT, alanine transaminase; AST, aspartate transaminase; CI, confidence interval; HR, hazard ratio; ID, iron deficiency; NT-proBNP, N-terminal pro-B-type natriuretic peptide; TSAT, transferrin saturation. | | | | | | |

| **Table S6. Cox Proportional Hazards Model for 15-year All-Cause Mortality (HF, n=182)** | | | | | | |
| --- | --- | --- | --- | --- | --- | --- |
|  | **Univariable Model** | | | **Multivariable Model *** | | |
|  | **HR** | **95% CI** | **P-value** | **HR** | **95% CI** | **P-value** |
| ID (heart failure guideline criteria) | 1.25 | 0.74, 2.11 | 0.4 | 1.16 | 0.60, 2.24 | 0.7 |
| Ferritin <300 ng/mL | 1.22 | 0.45, 3.30 | 0.7 | 1.45 | 0.69, 3.03 | 0.3 |
| Ferritin <100 ng/mL | 1.35 | 0.95, 1.92 | 0.10 | 1.20 | 0.70, 2.03 | 0.5 |
| Ferritin <30 ng/mL | 2.45 | 1.40, 4.30 | 0.002 | 2.64 | 1.40, 5.00 | 0.003 |
| Ferritin <15 ng/mL | 2.71 | 1.35, 5.44 | 0.005 | 2.72 | 1.02, 7.26 | 0.045 |
| TSAT <20% | 1.18 | 0.67, 2.08 | 0.6 | 1.07 | 0.62, 1.83 | 0.8 |
| Serum iron ≤13 μmol/L | 1.67 | 1.01, 2.76 | 0.045 | 0.97 | 0.60, 1.57 | 0.9 |
| Serum iron ≤10 μmol/L | 1.79 | 0.91, 3.52 | 0.093 | 1.12 | 0.62, 2.01 | 0.7 |
| ***** Model adjusted for age (/5), gender, race, body mass index, anemia, treatment for anemia, stroke, hypertension, diabetes, cancer, ln(ALT), ln(NT-proBNP), estimate glomerular filtration rate, percentage of neutrophils.  ALT, alanine transaminase; CI, confidence interval; ID, iron deficiency; HF, heart failure; HR, hazard ratio; NT-proBNP, N-terminal pro-B-type natriuretic peptide; TSAT, transferrin saturation. | | | | | | |

| **Table S7. Relationship of Dietary Intake to Iron Deficiency in General Population (Logistic Regression Model)** | | | | | | | | | | |
| --- | --- | --- | --- | --- | --- | --- | --- | --- | --- | --- |
|  |  | **Model 1** | | | **Model 2*** | | | **Model 3 †** | | |
| **Group** | **Dietary intake** | **OR** | **95% CI** | **P-value** | **OR** | **95% CI** | **P-value** | **OR** | **95% CI** | **P-value** |
| ID (heart failure guideline criteria) | Iron | 0.91 | 0.88, 0.95 | <0.001 | 0.94 | 0.91, 0.98 | 0.003 | 1.0 | 0.96, 1.03 | 0.8 |
|  | Total folate | 0.94 | 0.91, 0.97 | <0.001 | 0.97 | 0.94, 1.01 | 0.10 | 1.01 | 0.97, 1.05 | 0.7 |
| Ferritin <300 ng/mL | Iron | 0.97 | 0.96, 0.99 | <0.001 | 0.98 | 0.96, 0.99 | 0.013 | 0.99 | 0.97, 1.01 | 0.4 |
|  | Total folate | 0.99 | 0.98, 1.00 | 0.2 |  |  |  |  |  |  |
| Ferritin <100 ng/mL | Iron | 0.89 | 0.86, 0.93 | <0.001 | 0.92 | 0.88, 0.96 | 0.002 | 0.98 | 0.94, 1.02 | 0.3 |
|  | Total folate | 0.92 | 0.89, 0.95 | <0.001 | 0.95 | 0.91, 0.98 | 0.009 | 0.99 | 0.95, 1.03 | 0.5 |
| Ferritin <30 ng/mL | Iron | 0.95 | 0.92, 0.97 | <0.001 | 0.96 | 0.93, 0.99 | 0.019 | 1.00 | 0.97, 1.04 | 0.8 |
|  | Total folate | 0.97 | 0.95, 0.99 | 0.015 | 0.99 | 0.96, 1.01 | 0.3 | 0.99 | 0.96, 1.01 | 0.3 |
| Ferritin <15 ng/mL | Iron | 0.98 | 0.96, 1.0 | 0.014 | 0.98 | 0.96, 1.01 | 0.10 | 1.00 | 0.97, 1.03 | >0.9 |
|  | Total folate | 0.99 | 0.97, 1.01 | 0.2 |  |  |  |  |  |  |
| TSAT <20% | Iron | 0.96 | 0.92, 0.99 | 0.015 | 0.98 | 0.95, 1.02 | 0.3 | 1.01 | 0.97, 1.05 | 0.6 |
|  | Total folate | 0.96 | 0.93, 1.00 | 0.029 | 0.99 | 0.96, 1.03 | 0.7 | 1.01 | 0.97, 1.05 | 0.6 |
| Serum iron ≤13 μmol/L | Iron | 0.96 | 0.93, 0.99 | 0.018 | 0.99 | 0.96, 1.03 | 0.7 | 1.01 | 0.97, 1.05 | 0.5 |
|  | Total folate | 0.96 | 0.93, 1.00 | 0.053 | 1.00 | 0.96, 1.04 | 0.9 | 1.01 | 0.97, 1.06 | 0.5 |
| Serum iron ≤10 μmol/L | Iron | 0.97 | 0.94, 0.99 | 0.013 | 0.99 | 0.96, 1.02 | 0.3 | 1.00 | 0.97, 1.03 | 0.9 |
|  | Total folate | 0.96 | 0.94, 0.99 | 0.009 | 0.99 | 0.96, 1.02 | 0.4 | 1.00 | 0.97, 1.03 | 0.8 |
| ***** Model 2: Model adjusted for variables with P ≤0.1 in univariable model (excluding gender).  **†** Model 3: Model 2 + gender  CI, confidence interval; ID, iron deficiency; OR: odds ratio; TSAT, transferrin saturation. | | | | | | | | | | |

| **Table S8. Relationship of Dietary Intake to Iron Deficiency in HF Patients (Logistic Regression Model)** | | | | | | | | | | |
| --- | --- | --- | --- | --- | --- | --- | --- | --- | --- | --- |
|  |  | **Model 1** | | | **Model 2** | | | **Model 3** ‡ | | |
| **Group** | **Dietary intake** | **OR** | **95% CI** | **P-value** | **OR** | **95% CI** | **P-value** | **OR** | **95% CI** | **P-value** |
| Ferritin <30 ng/mL * | Iron | 1.03 | 0.86, 1.22 | 0.8 |  |  |  |  |  |  |
|  | Total folate | 1.19 | 1.03, 1.38 | 0.023 | 1.21 | 1.04, 1.41 | 0.015 | 1.21 | 1.05, 1.39 | 0.009 |
| Ferritin <15 ng/mL † | Iron | 1.06 | 0.99, 1.14 | 0.074 | 1.08 | 1.00, 1.16 | 0.050 | 1.08 | 1.00, 1.16 | 0.050 |
|  | Total folate | 1.06 | 0.98, 1.14 | 0.2 |  |  |  |  |  |  |
| ID (heart failure guideline criteria) | Iron | 0.98 | 0.74, 1.29 | 0.9 |  |  |  |  |  |  |
|  | Total folate | 0.93 | 0.70, 1.23 | 0.6 |  |  |  |  |  |  |
| Ferritin <300 ng/mL | Iron | 0.96 | 0.87, 1.06 | 0.4 |  |  |  |  |  |  |
|  | Total folate | 0.94 | 0.85, 1.04 | 0.2 |  |  |  |  |  |  |
| Ferritin <100 ng/mL | Iron | 1.02 | 0.79, 1.32 | 0.9 |  |  |  |  |  |  |
|  | Total folate | 0.91 | 0.74, 1.13 | 0.4 |  |  |  |  |  |  |
| TSAT <20% | Iron | 0.96 | 0.74, 1.24 | 0.7 |  |  |  |  |  |  |
|  | Total folate | 1.04 | 0.79, 1.37 | 0.7 |  |  |  |  |  |  |
| Serum iron ≤13 μmol/L | Iron | 1.09 | 0.85, 1.40 | 0.5 |  |  |  |  |  |  |
|  | Total folate | 1.09 | 0.83, 1.42 | 0.5 |  |  |  |  |  |  |
| Serum iron ≤10 μmol/L | Iron | 1.13 | 0.88, 1.44 | 0.3 |  |  |  |  |  |  |
|  | Total folate | 1.16 | 0.92, 1.47 | 0.2 |  |  |  |  |  |  |
| * Model 2 (ferritin <30 ng/mL): Model adjusted for ln (ALT), glycohemoglobin, treatment for anemia, hospitalization, anemia, NYHA functional class.  † Mocel 2 (ferritin <15 ng/mL): Model adjusted for NYHA functional class, hemoglobin.  ‡ Model 3: Model 2 + gender  ALT, alanine transaminase; CI, confidence interval; HF, heart failure; ID, iron deficiency; NYHA, New York Heart Association; OR, odds ratio; TSAT, transferrin saturation. | | | | | | | | | | |
